# Amino Surface Modification and Fluorescent Labelling of Porous Hollow Organosilica Particles: Optimization and Characterization

**DOI:** 10.3390/ma15072696

**Published:** 2022-04-06

**Authors:** Mohammed A. Al-Khafaji, Anikó Gaál, Bálint Jezsó, Judith Mihály, Zoltán Varga

**Affiliations:** 1 Research Centre for Natural Sciences, Institute of Materials and Environmental Chemistry, H-1117 Budapest, Hungary; al-khafaji.mohammed@ttk.hu (M.A.A.-K.); gaal.aniko@ttk.hu (A.G.); jezso_balint@hotmail.com (B.J.); mihaly.judith@ttk.hu (J.M.); 2 Hevesy György PhD School of Chemistry, Eötvös Loránd University, H-1117 Budapest, Hungary

**Keywords:** hollow silica particles, aminopropyl surface modification, fluorescent labelling

## Abstract

Surface modification of silica nanoparticles with organic functional groups while maintaining colloidal stability remains a synthetic challenge. This work aimed to prepare highly dispersed porous hollow organosilica particles (pHOPs) with amino surface modification. The amino-surface modification of pHOPs was carried out with 3-aminopropyl(diethoxy)methylsilane (APDEMS) under various reaction parameters, and the optimal pHOP-NH_2_ sample was selected and labelled with fluorescein isothiocyanate (FITC) to achieve fluorescent pHOPs (F-HOPs). The prepared pHOPs were thoroughly characterized by transmission electron microscopy, dynamic light scattering, FT-IR, UV-Vis and fluorescence spectroscopies, and microfluidic resistive pulse sensing. The optimal amino surface modification of pHOPs with APDEMS was at pH 10.2, at 60 °C temperature with 10 min reaction time. The positive Zeta potential of pHOP-NH_2_ in an acidic environment and the appearance of vibrations characteristic to the surface amino groups on the FT-IR spectra prove the successful surface modification. A red-shift in the absorbance spectrum and the appearance of bands characteristic to secondary amines in the FTIR spectrum of F-HOP confirmed the covalent attachment of FITC to pHOP-NH_2_. This study provides a step-by-step synthetic optimization and characterization of fluorescently labelled organosilica particles to enhance their optical properties and extend their applications.

## 1. Introduction

Porous hollow organosilica particles (pHOPs), among several silica nanoparticles, have attracted researchers’ attention in many fields due to their unique and functional properties. Their large encapsulated inner space and their porous shell framework gain these particles a larger specific surface area and higher concentration of free silanol groups on the surface than solid silica nanoparticles. Accordingly, these particles have many features that are a general platform for functionalized silica nanomaterials [1,2]. The surface modification of pHOPs with amino groups is an essential strategy for more accessible surface decorations with other functional groups (such as fluorescent markers, contrast agents and other biomolecules), which could expand the diversity in their applications [3,4]. Two conventional approaches for surface modification are usually proposed: post-grafting and direct condensation [5,6]. Each has its drawbacks and advantages. For example, in the post-grafting process, the organic moieties have a non-homogeneous distribution on the surface of nanoparticles, mainly near the inner surface of the pores, which it sometimes transcends to completely block the pores when the concentration of functional moieties is high. In the direct condensation approach, the organic moieties have a homogeneous distribution in the entire silica framework without causing pore-enclosed [5,7]. The direct condensation process suffers from the less functional groups located at the surface and the low stability of the silica structure. This limitation can affect the concentration of organic functional groups on the silica surface because the higher the concentration of functional groups on the surface, the more unstable the silica structure [8,9]. Apart from the direct condensation process, the original structure of the starting materials is usually preserved after surface modification via post-grafting [10].

Generally, the surface modification of pHOPs via the post-grafting strategy can be accomplished by two steps: First, native pHOPs are prepared, followed by covalently attaching organic functional groups to the free silanol (Si-OH) on the surface via the Si-O-Si linkage. It is widely common that the hydrolyzed R-Si-OH groups can undergo self-condensation to form dimers and trimers. Thus, by-products can be formed due to the self-condensation of these dimers and trimers or cross-linking with neighbouring particles causing particle aggregates, which affects the colloidal stability and limits their applications [11]. These problems can be overcome by controlling the incorporation of functional groups on the surface of the substrate. Several attempts have been employed to study the influence of surface charge on colloidal stability [12,13,14]. For example, Bini and co-workers studied the influence of the number of amino groups on the surface on colloidal stability. The results indicated that the surface charge and the stability of colloid dispersity could be tuned by controlling the concentration of amino groups on the surface [15].

Based on the discussion above, there is a conflict between using an excess concentration of functional groups to increase the total number of active sites on the surface and the colloidal stability. Alternatively, decreasing the concentration of the functional groups prevents unwanted products and limits the total number of active sites on the surface. To avoid self-condensation and to enhance the grafting process, the reaction parameters should be precisely controlled [7,12,16,17]. In the present work, amino-modified pHOPs were synthesized via the post-grafting approach at different reaction parameters. The influence of the reaction parameters such as pH, temperature, reaction time, and 3-aminopropyl(diethoxy)methyl silane (APDEMS) concentration on the grafting process was further explored by using dynamic light scattering (DLS) and Zeta potential measurements. The reaction parameters were adjusted one by one to obtain pHOPs-NH_2_ with high colloidal stability and a maximum number of amino groups on the surface. The presence of surface amino groups was confirmed by infrared spectroscopy (IR). Finally, the amino-modified pHOPs were fluorescently labelled with fluorescein isothiocyanate (FITC). The successful synthesis of fluorescent pHOPs was confirmed by UV–Vis and fluorescent spectroscopies. The monodispersity of such particles is of great importance. Therefore, we have also utilized microfluidic resistive pulse sensing (MRPS) to characterize the size distribution and concentration of the F-HOP sample. As we have shown in our previous study on the characterization of bimodal solid silica nanoparticles [18], the use of orthogonal techniques (i.e., optical and non-optical methods) for the measurement of the size distribution is critically important. The number of FITC molecules per particle was calculated based on concentration measurements by MRPS and fluorescence spectroscopy measurements.

## 2. Materials and Methods

### 2.1. Reagents

Tetraethylorthosilicate (TEOS, reagent grade, Sigma-Aldrich, Budapest, Hungary), ammonium hydroxide (NH_4_OH, 28–30%, MACRON), ethanol (EtOH, ACS, MACRON), hexadecyltrimethylammonium bromide (CTAB, >98%), 3-Aminopropyl(diethoxy)methylsilane (APDEMS, 97%), hydrochloric acid (HCl, ACS reagent 37%, Reanal, Budapest, Hungary), glacial acetic acid (EMSURE, Merck, Darmstadt, Germany), 1,2-bis(triethoxysilyl)ethane (BTEE, 96%, Sigma-Aldrich, Budapest, Hungary), fluorescein isothiocyanate (FITC, ≥95% spectrophotometric assay, Sigma-Aldrich, Budapest, Hungary), and sodium carbonate (Na_2_CO_3_, >99.5%, Reanal, Budapest, Hungary), were used as purchased without further purification.

### 2.2. Sample Preparation

#### 2.2.1. Synthesis of Porous Hollow Organosilica Particles (pHOPs)

Monodisperse pHOPs were synthesized using a pre-synthesized solid SiO_2_ core as a hard template. The solid SiO_2_ core with a particle size of 280 nm in diameter was first synthesized as follows: 6 mL TEOS was rapidly added to a mixture of 20 mL Milli-Q water, 60 mL absolute ethanol, and 7.5 mL ammonia solution, with constant stirring at 500 rpm at 30 °C. The reaction was maintained for one hour before the resulting sSiO_2_ particles were collected by centrifugation at 5000× *g* for 10 min at room temperature in Falcon™ 50 mL conical centrifuge tubes and washed twice with Milli-Q water and twice with ethanol. Finally, the collected sSiO_2_ particles were dried under vacuum overnight. Next, core-shell particles were synthesized via a co-condensation mixture of TEOS and BTEE as inorganic/organic silica precursors on the surface of the sSiO_2_ core in the presence of CTAB as a pore-forming agent. In a typical procedure, 100 mg of sSiO_2_ core was resuspended in 20 mL ultrapure water and sonicated for 1 h (ELMASONIC S10, Elma Ultrasonic Technologies, Singen, Germany).

In parallel, 180 mg of CTAB surfactant was dissolved in a 30 mL 1:1 aqueous ethanol solution containing 1.1 mL ammonia. The CTAB solution was stirred for 30 min before the colloidal sSiO_2_ solution was slowly added dropwise under vigorous stirring. Afterwards, 50 μL BTEE was added immediately after adding 10 μL TEOS to the above mixture. The reaction mixture was maintained for 6 h before the resulting core-shell particles were collected by centrifugation at 5000× *g* for 10 min at room temperature in Falcon™ 50 mL conical centrifuge tubes and washed twice with ethanol and twice with Milli-Q water. For the sSiO_2_ core removal, the above core-shell particles were resuspended in a 12.5 mL Milli-Q water containing 787.5 mg of Na_2_CO_3_ and stirred at 80 °C for 1 h. The resulting pHOPs were collected by centrifugation and washed twice with Milli-Q water and twice with ethanol. Finally, the resulting product was resuspended in 10 mL of ethanol solution containing 10% concentrated HCl and stirred at 60 °C for 3 h to remove the surfactant. The last step was repeated three times to ensure complete removal of the CTAB. The final product was resuspended in 20 mL ethanol.

#### 2.2.2. Synthesis of Amino-Modified pHOPs (pHOP-NH_2_)

The surface functionalization of pHOPs with amino groups was performed via the post-grafting approach in ethanol solution at different reaction parameters. 3-aminopropyl(diethoxy)methyl silane (APDEMS) was used as the surface modifier. The reaction parameters were evaluated one by one as follows: 0.5 mL of the above pHOP sample was transferred into a 4 mL vial containing 2 mL absolute ethanol. The pH of the mixture was adjusted to the desired value with acetic acid or ammonia. The reaction mixtures were stirred at 500 rpm at the desired reaction temperature. Next, the required amount of a freshly prepared stock solution of 13 μL of APDEMS in 1 mL absolute ethanol was quickly added to the above solution. The reaction mixtures were maintained for the desired time. The resulting pHOP-NH_2_ product was collected by centrifugation at 5000× *g* for 10 min using 2 mL Eppendorf™ Snap-Cap microcentrifuge tubes and washed with ethanol four times to ensure complete removal of the residual unreacted APDEMS. Finally, the pHOP-NH_2_ were resuspended in 2 mL ethanol. All reaction conditions related to this experiment are listed in Table 1.

#### 2.2.3. Fluorescent Labelling of pHOP-NH_2_ with FITC

For the fluorescent labelling of pHOP-NH_2_, 0.01 M of FITC dye solution in ethanol was prepared in an amber, pre-weighed glass vial. The solution was continuously stirred for 30 min to ensure the dye’s complete dissolution. A fourfold excess of FITC solution (497.8 μL of 0.01 M) compared to the initial APDEMS concentration was added into a 4 mL amber glass vial containing 2 mL of pHOP-NH_2_ sample. The reaction mixture was stirred overnight at room temperature under dark conditions to ensure a complete reaction between the surface amino groups with FITC molecules. The resulting F-HOP product was collected by centrifugation at 5000× *g* for 10 min using a 2 mL Eppendorf™ Snap-Cap microcentrifuge tube and washed with ethanol. The last step was repeated four times until FITC was not detectable in the supernatant when using fluorescent spectroscopy. The collected sample was resuspended in 2 mL absolute ethanol and kept in the fridge at 4 °C. The supernatant of the washing steps was pooled and analyzed by fluorescence spectroscopy to calculate the residual amount of unreacted FITC.

### 2.3. Sample Characterization

#### 2.3.1. Transmission Electron Microscopy (TEM)

TEM images were collected with a JEM 1011 (JEOL Ltd., Tokyo, Japan) transmission electron microscope operated at an accelerating voltage of 80 kV. For preparation, 3 μL from the sample was dropped on a Formvar-coated, 200 mesh copper grid for 60 s. Next, the excess sample was blotted using filter paper. All images were analyzed using ImageJ (Version 1.46, National Institute of Mental Health, Bethesda, MD, USA). Mean diameter and standard deviation (SD) for more than 100 particles were calculated by fitting a Gaussian function to the measured size distribution in Origin (OriginPro 2018, OriginLab Corporation, Northhampton, MA, USA).

#### 2.3.2. Dynamic Light Scattering (DLS)

DLS measurements were carried out on a W130i DLS system (AvidNano, High Wycombe, UK), equipped with a laser diode (660 nm) and an avalanche photodiode detector located at a side-scattering angle of 90°. The measurements were collected at a controlled temperature of 20 ± 0.2 °C using low-volume disposable plastic cuvettes (UVette, Eppendorf Austria GmbH, Wien, Austria). Intensity autocorrelation functions were analyzed with the iSize 3.0 software (AvidNano, High Wycombe, UK), utilizing a continuous I (D) distribution model (CONTIN algorithm). Mean hydrodynamic diameter values and intensity size distributions are reported as provided by the iSize software.

#### 2.3.3. Zeta Potential Measurements

Zeta potential measurements were carried out on a Zetasizer Nano ZS (Malvern Instruments Ltd., Malvern, UK) instrument. The data were collected in triplicate at room temperature (~25 °C) using a disposable folded capillary tube cell. The pH was adjusted by mixing 60 μL from the synthesized sample with 600 μL of pre-titrated HCl or NaOH solution at the desired pH.

#### 2.3.4. Microfluidic Resistive Pulse Sensing (MRPS)

MRPS measurements were performed with an nCS1 instrument (Spectradyne LLC, Signal Hill, CA, USA). The F-HOP sample was first diluted 10-fold in Milli-Q water, followed by another 10-fold dilution in 0.6 mM sodium dodecyl sulfate (SDS, Sigma-Aldrich, Budapest, Hungary) in phosphate-buffered saline solution (PBS, Sigma-Aldrich, Budapest, Hungary) filtered through an Amicon Ultra, 100 kDa MWCO membrane filter (Sigma-Aldrich, Budapest, Hungary). A factory calibrated C-900 cartridge with 130 nm to 900 nm measurement range was used for the measurements.

#### 2.3.5. Attenuated Total Reflection Fourier-Transform Infrared Spectroscopy (ATR-FTIR)

ATR-FTIR spectra were recorded from 4000 to 400 cm^−1^ using a Varian 2000 (Scimitar Series) FTIR spectrometer (Varian Inc., Palo Alto, CA, USA) equipped with a broadband MCT detector and a single reflection diamond ‘Golden Gate’ ATR accessory (Specac Ltd., Orpington, UK). For the measurements, 3 μL of the sample was dropped on the diamond ATR surface and dried under inert nitrogen flow. Sixty-four scans were collected at a resolution of 2 cm^−1^, keeping the sample under an inert nitrogen atmosphere during the measurement.

#### 2.3.6. UV-Vis Spectroscopy

UV-visible spectra were recorded using a Hewlett Packard 8453 UV-Vis spectrophotometer (Hewlett Packard, Palo Alto, CA, USA). Measurements were performed using a quartz micro-cuvette (1 mm × 1 cm) in the wavelength range of 250 nm to 800 nm with 1 nm resolution at room temperature.

#### 2.3.7. Fluorescence Spectroscopy

Fluorescence spectroscopy measurements were carried out using an SP-8500 (JASCO International Co., Ltd., Tokyo, Japan) spectrofluorometer. Measurements were carried out at a controlled temperature of 20 ± 0.2 °C using a quartz micro-cuvette with a path length of 3 mm, at a 400 nm/min scan speed. A calibration curve using free FITC solution at different concentrations was used to estimate the amount of unreacted FITC after labelling.

#### 2.3.8. Small-Angle X-ray Scattering (SAXS)

The porosity of the final F-HOP sample was investigated with SAXS. Measurements were performed using the CREDO SAXS instrument [19]. The sample was filled into a borosilicate glass capillary with a nominal diameter of 1.5 mm (Hilgenberg GmbH, Malsfeld, Germany), and the 2D scattering patterns were collected at a sample-to-detector distance of 529 mm with a Pilatus-300k CMOS hybrid pixel detector (Dectris Ltd., Baden, Switzerland). Data analysis was performed with the instrument’s software package described elsewhere [19].

## 3. Results and Discussion

**Figure 1:**
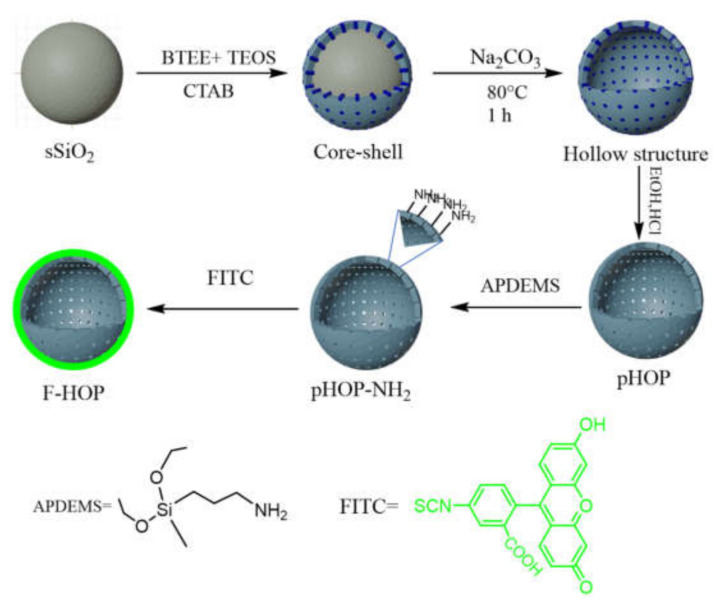
Schematic representation for the preparation of amino-modified pHOPs and F-HOPs.

The focus of this study is to gain a better understanding of the influence of reaction parameters on the surface modification of porous organosilica particles. Our goal was to reach the highest possible concentration of amino groups on the surface of pHOPs while maintaining its colloidal stability. The study was extended to include the fluorescent labelling of amino-modified pHOPs with FITC fluorescent dye. The synthesis strategy of this work is illustrated in Figure 1. First, solid silica core particles (sSiO_2_) were prepared by the Stöber method, which was used as the template for the synthesis of core-shell particles by co-condensation of tetraethyl orthosilicate (TEOS) and 1,2-bis(triethoxysilyl) ethane (BTEE) on their surface in the presence of CTAB as a pore-forming agent. Hollow particles (pHOPs) were achieved by removing the inner core by basic hydrolysis in 0.6 M Na_2_CO_3_ solution at 80 °C. In the next step, the amino-surface modification of pHOPs was carried out under various reaction parameters and, finally, the optimal pHOP-NH_2_ sample was selected and labelled with FITC to achieve fluorescently labelled F-HOP particles.

### 3.1. Size and Morphology of Unmodified pHOPs

**Figure 2:**
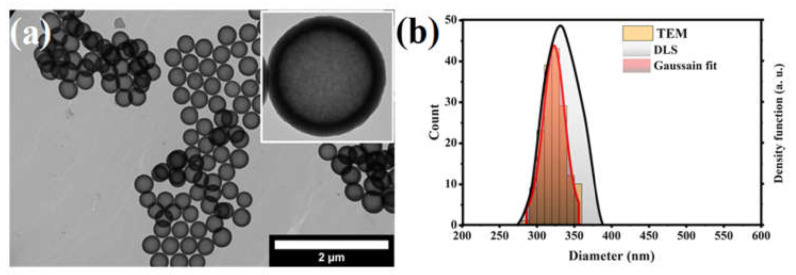
TEM image of the unmodified pHOP sample (**a**), and the particle size distributions of pHOPs based on TEM and DLS (**b**).

The morphology and size of the solid silica core and pHOPs samples were characterized by TEM and DLS. According to these results, the solid SiO_2_ particles have a uniform spherical morphology with 280 nm particle diameter and 38 nm standard deviation (Figure S1). Figure 2. shows the TEM and DLS characterization of pHOPs. TEM revealed highly uniform particles with a hollow structure and a spherical shape. The average outer particle diameter and shell thickness for the pHOPs were found to be 322 nm and 33 nm, respectively, based on the analysis of the TEM images (Figure S2. The mean hydrodynamic diameter and polydispersity index (PDI) of unmodified pHOPs was determined by DLS and found to be 331 nm and 0.5, respectively.

### 3.2. Optimizing the Reaction Conditions for Preparing Amino-Modified pHOPs (pHOP-NH_2_)

**Figure 3:**
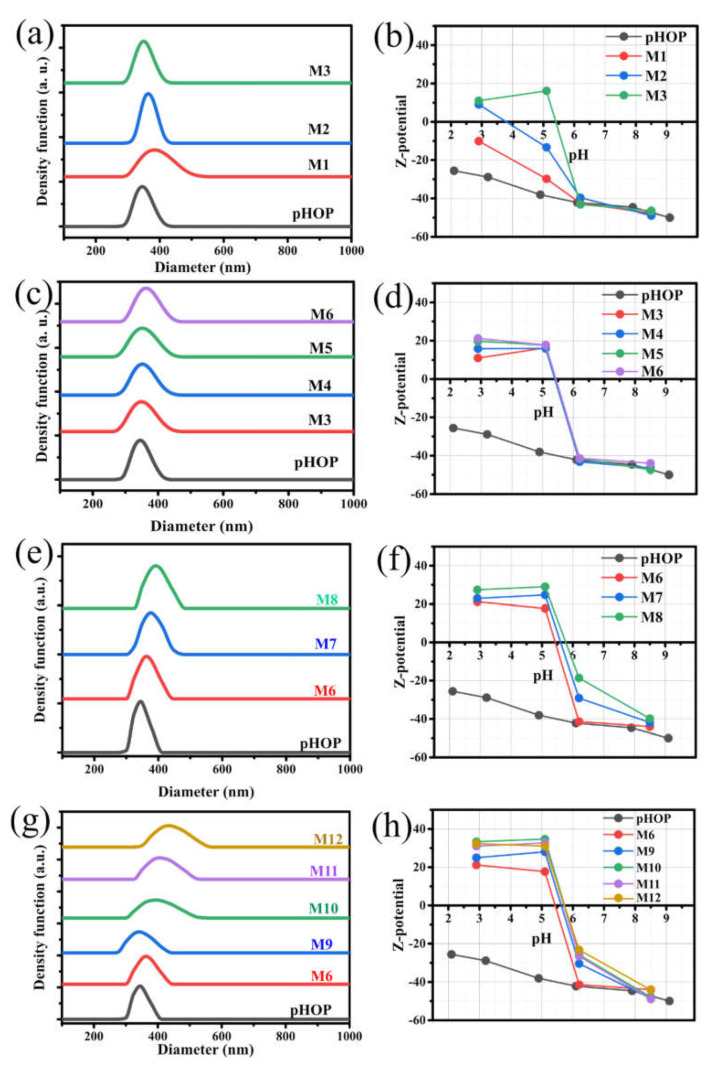
Intensity weighted size distributions from DLS measurements and Zeta potential values of pHOP and pHOP-NH_2_ samples prepared at (**a**,**b**) different pH environments, acidic (M1), medium (M2), and basic (M3), (**c**,**d**) different reaction temperatures, 30 (M3), 40 (M4), 50 (M5), and 60 °C (M6), (**e**,**f**) different reaction times 10 (M6), 30 (M7), and 60 min (M8) and (**g, h**) different APDEMS concentrations, 0.6 (M6), 1.2 (M9), 1.8 (M10), 2.4 (M11) and 3.0 μmol (M12).

The influences of reaction parameters (such as pH, temperature, reaction time and APDEMS concentration) on the properties of pHOP-NH_2_ samples were systematically investigated using DLS and Zeta potential measurements. Therefore, a series of pHOPs-NH_2_ samples at different reaction parameters were synthesized via post-grafting of APDEMS on the surface of pHOPs. The parameters corresponding to each sample are summarized in Table 1. DLS and Zeta potential measurement results at four different pH values of all prepared pHOP and pHOP-NH_2_ samples are summarized in Table 2. Figure 3 shows the intensity weighted particle size distributions (PSDs) of the pHOPs-NH_2_ samples from DLS measurements and the Zeta potential of the particles as the function of pH.

#### 3.2.1. Effect of pH

Figure 3a shows the size distributions by DLS for unmodified pHOPs and amino-modified pHOPs synthesized at acidic, neutral and basic reaction environments. While a broad PSD can be observed for the pHOP-NH_2_ sample prepared at acidic reaction environment (M1), the pHOP-NH_2_ samples prepared at neutral (M2) and basic (M3) reaction environments result in particles with narrow PSDs. Figure 3b shows the Zeta potential measurements at different pH values for the unmodified pHOP and pHOP-NH_2_ samples synthesized at different pH environments. The negative Zeta potential of unmodified pHOP at the whole pH range is attributed to the deprotonation of the free silanol groups on the surface of pHOPs. A remarkable shift in Zeta potential at acidic pH (pH = 2.9) from negative to positive values was observed after amino surface modification. The positive shift in the Zeta potential is attributed to the protonation of amino groups on the surface of the particles. Furthermore, the larger the positive charge of the particles, the more amino groups there are on the surface. In addition, the isoelectric point (IEP), the pH value where the surface charge of the modified particles is equal to zero, was determined. The increase in Zeta potential at pH 2.9 and IEP for pHOP-NH_2_ samples during the transition from acidic to neutral and basic reaction environments indicates that more amino groups are presented on the surface of pHOPs. These results suggest that the grafting process is mainly dependent on the pH of the reaction environment. The broader PSD and the lower positive charge of the pHOP-NH_2_ prepared at the acidic environment (M1) indicate that it is not preferable for the surface modification of pHOPs with APDEMS. On the contrary, the pHOP-NH_2_ sample prepared at the neutral (M2) and basic environment (M3) has shown narrow PSDs with higher Zeta potential and IEP values. These observations indicate that the grafting process is preferable in neutral and most preferable in the basic reaction environment. Accordingly, the basic reaction environment was chosen as the optimal reaction condition.

#### 3.2.2. Effect of Reaction Temperature

Four different reaction temperatures (30, 40, 50, and 60 °C) have been applied to modify pHOPs with APDEMS at a basic reaction environment to study the effect of the reaction temperature on the surface modification process. Figure 3c shows the DLS size distributions for unmodified pHOP and pHOP-NH_2_ samples prepared at different reaction temperatures, while Table 2 contains the corresponding mean diameter and PDI values. The narrow PSDs and slightly increasing mean diameter values can be observed for all pHOP-NH_2_ prepared at different reaction temperatures, suggesting that pHOP-NH_2_ samples do not aggregate when the reaction temperature increases from 30 up to 60 °C. Figure 3d displays the Zeta potential measurements at different pH values for unmodified pHOP and pHOP-NH_2_ samples prepared at different reaction temperatures. The Zeta potential values for pHOP-NH_2_ prepared at 30 (M3), 40 (M4), 50 (M5) and 60 C (M6) at pH 2.9 were found to be 11, 13.4, 19.7, and 21.2 mV, respectively. All samples have almost similar IEP values of ~5.4. The increase in the positive charge of pHOP-NH_2_ samples with increasing reaction temperature can be attributed to the increasing of the number of grafted amino groups on the surface of pHOPs. The above results indicate that the pHOP-NH_2_ synthesized at 60 °C has a narrow PSD and a higher Zeta potential than the samples synthesized at lower temperatures. Hence, 60 °C was selected as an optimal reaction temperature.

#### 3.2.3. Effect of Reaction Time

The third factor in our study that influences the surface modification of pHOPs is the reaction time. Figure 3e shows the DLS size distributions for unmodified pHOP and pHOP-NH_2_ samples prepared by applying 10, 30 and 60 min reaction times, while Table 2 contains the corresponding mean diameter and PDI values. Prolonging the reaction time causes a slight shift of the mean diameters to larger values. In parallel, Zeta potential values for the pHOP-NH_2_ samples prepared at different reaction times increased from 21.2 to 22.9 and 27.4 mV, and IEP was also found to increase with longer reaction times. The results mentioned above indicate that the 10 min reaction time was sufficient to occupy most of the free silanol groups on the surface of pHOPs with APDEMS molecules. However, when the reaction time is long enough to reach the equilibrium point, the particle aggregates become more likely and affect the colloidal stability. Thus, 10 min reaction time was considered as the optimal parameter.

#### 3.2.4. Effect of APDEMS Concentration

The effect of the concentration on the surface modifier on the colloidal stability was investigated at 0.6 (M6), 1.2 (M9), 1.8 (M10), 2.4 (M11) and 3.0 μmol (M12) APDEMS amounts. Figure 3g shows the DLS size distributions for unmodified pHOP and pHOP-NH_2_ samples prepared at different concentrations of APDEMS, while Table 2 contains the corresponding mean diameter and PDI values. As observed on the PSDs, APDEMS amounts of 1.8 μmol or higher causes the shift of the mean diameters to larger values and significant broadening of the distributions indicating particle aggregation. Zeta potential values at pH 2.9 were 25.4, 33.3, 31, and 32.3 mV for M9, M10, M11, and M12, respectively. The highest positive surface charge was obtained when the molarity of APDEMS was 1.8 μmol (M10). A slight decrease in surface positive charge values beyond 1.8 μmol of APDEMS suggests that the silanol groups on the surface are saturated with APDEMS molecules, and the modification process reaches the highest degree.

Although the Zeta potential measurements for the M10 sample showed the highest positive charge, it also showed a poor particle size distribution based on the DLS. Accordingly, the reaction parameters used to prepare the M9 sample with a high positive charge value and narrow size distribution have been chosen as the optimal reaction conditions.

### 3.3. Fluorescent Labelling of pHOP-NH_2_ with FITC

#### 3.3.1. Size, Morphology, and Zeta Potential of F-HOPs

**Figure 4:**
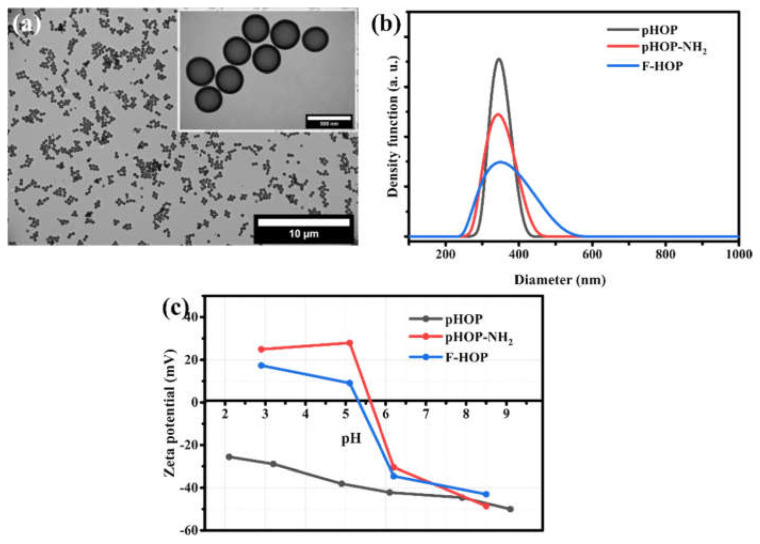
TEM image of F-HOPs (**a**), and size distributions by DLS (**b**) and Zeta potentials as the function of pH (**c**) of pHOP, pHOP-NH_2_, and F-HOP samples.

The incorporation of fluorescence isothiocyanate (FITC) on the surface of pHOP-NH_2_ to form fluorescent porous hollow organosilica particles (F-HOPs) was performed in ethanol solution at room temperature under dark conditions. The primary amino groups on the surface of pHOP-NH_2_ particles were covalently coupled with CSN-functional groups of FITC dye via thiourea bonds. This covalent incorporation of FITC onto the pHOPs could minimize the FITC dye leaching [20,21]. The size and morphology of synthesized F-HOPs were characterized by TEM. The TEM image of F-HOPs (Figure 4a) shows similar morphology to that of unmodified pHOPs (Figure 2a). Monodisperse PSD and uniform shell thickness were also observed after fluorescent labelling, revealing that the hollow structure of these particles is preserved through the modifications process. The average diameter and shell thickness of F-HOPs determined from their particle size distribution (Figure S3) were 322 and 34 nm, respectively. DLS indicated a mean diameter of F-HOPs near that of the unmodified pHOPs (326 nm), but the polydispersity of the distribution increased. The Zeta potential in acidic medium (pH 2.9) of the F-HOP sample decreased to 17.4 mV from 25.4 mV of the pHOP-NH_2_, which indicates the reduction of free amino groups on the surface of the particles in line with the covalent coupling of FITC to the surface.

#### 3.3.2. FT-IR Spectroscopy of pHOP, pHOP-NH_2_, and F-HOP

**Figure 5:**
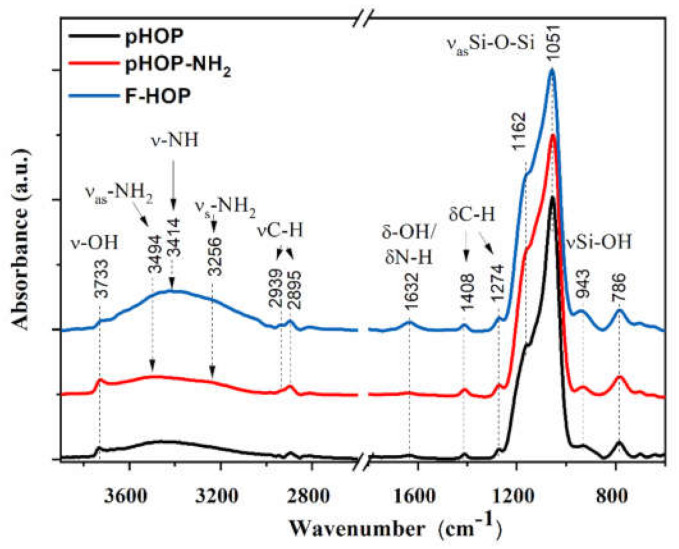
FT-IR spectra of unmodified pHOP (black line), pHOP-NH_2_ (red line) and F-HOP (blue line) samples. Spectra are normalized to the strongest Si-O-Si band intensity and are shifted vertically for better visualization.

FT-IR spectroscopy was employed to confirm the successful immobilization of amino groups on the surface of the pHOPs, and then fluorescent labelling. Figure 5 compares the IR spectra of unmodified pHOPs, followed by amine surface modification (pHOP-NH_2_) and finally decorated by an FITC fluorescent label (F-HOP). All spectra are dominated by the strong absorptions of Si-O-Si exhibiting bands around 1051 cm^−1^ (ν_as_Si-O-Si) and 786 cm^−1^ (ν_s_ Si-O-Si). Surface –OH groups are represented by the –OH and Si-OH stretching bands at 3733 and 943 cm^−1^, respectively [22,23,24]. The organic ethylene-bridges in the network of the shell framework show C-H bands in the 3000–2800 cm^−1^ region [25], together with the C-H bending from Si-CH_2_-CH_2_-Si moieties of the organosilica shell at 1408 and 1274 cm^−1^, respectively. Only slight spectral modifications can be witnessed after surface modification of pHOPs with amino groups (HOP-NH_2_). The presence of Si-OH bands (3733 and 943 cm^−1^) suggests that unreacted silanol groups are still present on the surface. However, two shoulders can be clearly identified on the broad –OH stretching envelope around 3350 cm^−1^, resulting from the antisymmetric and symmetric stretching of primary amines [26]. The shoulders at 3494 cm^−1^ (ν_as_-NH_2_) and 3256 cm^−1^ (ν_s_-NH_2_) confirm the presence of surface amine groups. N-H bending vibrations [27,28] are unhappily masked by the –OH deformation bands around 1632 cm^−1^. By anchoring FITC to the surface amines, a new band is emerging in the N-H stretching region. This band around 3414 cm^−1^ can be assigned to the N-H stretching of secondary amines. Indeed, upon FITC anchoring, the N=C=S group of the label molecule transforms into the –NH-C(=S)-NH- moiety. The resonant –NH-C-NH-structure might lead to the enhanced intensity of the –NH stretching. A slight intensity increase of the –OH deformation band is also witnessed. FITC molecules, anchored to the surface of pHOPs, have also -OH groups, which might make a slight contribution to the intensity of this band. Moreover, the conjugated C=C bonds from FITC may exhibit a band around 1620–1640 cm^−1^, which again adds to the band intensity at 1632 cm^−1^.

#### 3.3.3. Optical Properties of F-HOPs

**Figure 6:**
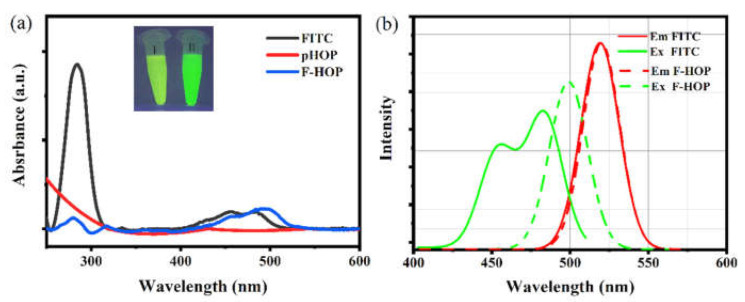
(**a**) UV-Vis absorption spectra of free FITC (black), pHOP (red), and F-HOP samples (blue). The inset shows the photograph of FITC solution (I) and the F-HOP sample (II) under UV light (385 nm). (**b**) Fluorescent excitation (green) and emission (red) spectra for free FITC (solid) and F-HOP (dashed) samples measured in ethanol.

The optical properties of the F-HOP sample were investigated by UV-Vis and fluorescence spectroscopies. Figure 6a shows the UV-Vis absorption spectrum of the F-HOP compared with the spectra of unmodified pHOP and free FITC. Whereas the pHOP’s spectrum (Figure 6a red line) showed no absorbance peak, the F-HOP sample exhibited absorbance peaks at 285 and 488 nm (Figure 6a blue line), which are consistent with the spectrum of FITC (Figure 6a black line). Moreover, the red-shift in the characteristic absorbance peak accompanied by a shoulder at around 500 nm indicates that the FITC molecules are chemically coupled with amino groups on the surface.

The fluorescence excitation spectra of free FITC and F-HOP measured at the 520 nm emission wavelength resemble their absorption spectra. Fluorescent spectra of FITC and F-HOP (Figure 6b) show that λ_ex_ changed from 483 nm to 499 nm, while λ_em_ remained unchanged after coupling FITC to the surface of pHOP-NH_2_. Photographs of the free FITC solution (I) and the F-HOP sample (II) taken under long-wavelength UV light (385 nm) also confirm the chemical coupling of the dye (Inset of Figure 6a). This decrease in the Stokes shift also indicates the covalent attachment of the fluorophore to the organosilica surface.

**Figure 7:**
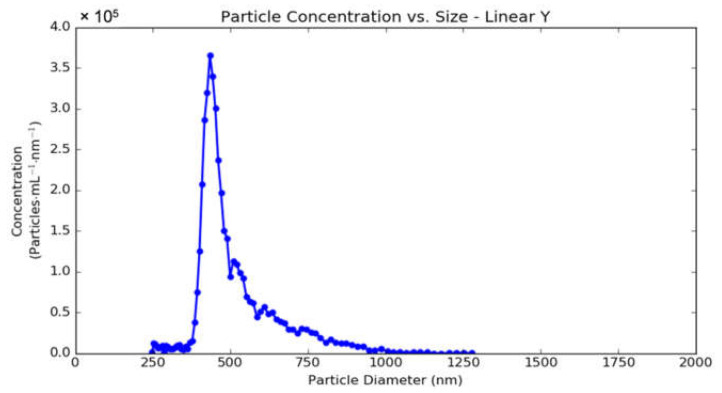
The concentration density function of the F-HOP sample as determined by MRPS. A 100-fold dilution was applied to meet the measurement range of the instrument. The total concentration of the particles as calculated from the area under the curve was 4.26 × 10^9^ mL^−1^.

The average number of FITC molecules on the surface of pHOPs was calculated by determining the total concentration of FITC coupled to the particle’s surface divided by the number concentration of the particles. The latter was determined by microfluidic resistive pulse sensing (MRPS). Figure 7 shows the concentration density function of the F-HOP sample. The area under the curve equals the total concentration of the particles, which was found to be 4.26 × 10^9^ mL^−1^. Besides the total concentration, the MRPS distribution also confirms that the F-HOP sample has a monomodal size distribution in agreement with previous TEM and DLS experiments.

The total FITC concentration was determined by measuring the unreacted FITC concentration in the washing steps’ supernatant by fluorescence spectroscopy and subtracting the concentration thus determined from the known initial concentration. For this purpose, fluorescent emission spectra of a series of known concentrations of FITC solution were measured and a calibration curve was constructed (Figure S4). This calculation yields 4 × 10^−5^ M for the particle-bound FITC. This value divided by the number concentration of particles yields 5.6 × 10^6^ molecules of FITC per particle. However, it should be kept in mind that the as determined number of fluorophores per particle does not necessarily result in the fluorescence intensity of a given number of pure fluorochrome molecules in solution (MESF). For the latter, further flow cytometry experiments are needed, which is out of the scope of the current manuscript.

#### 3.3.4. Characterization of the Porous Structure of F-HOP with SAXS and Its Colloidal Stability with DLS

SAXS was applied to prove the porous structure of the final F-HOP sample. Figure S5 shows the scattering curve of the sample. The peak at *q* = 1.586 nm^−1^ indicates a well-ordered porous structure. This peak position corresponds to a hexagonal lattice parameter of $a=2d/\sqrt{3}=4.575$ nm (where $d=2\pi /q_{\mathrm{peak}}$), which is in good agreement with literature data on MSN-41 type silica particles, indicating that F-HOP sample resembles these type of silica particles regarding their porosity [29,30].

The long-term stability of the F-HOP sample was investigated with DLS. Three months after the preparation, the measurement indicated a monodisperse sample with an average hydrodynamic diameter corresponding to the initial sample (Figure S6). It should be mentioned that this result only proves the stability in water. Stability in a more complex media may require further surface modification (e.g., PEGylation) [31]. Furthermore, the stability test by DLS only proves the particles’ morphological stability, and it does not necessarily prove the chemical stability of the amino surface modification. Indeed, Etienne and coworkers described the decreased stability of amino-functionalized silica materials in an aqueous media [32,33]. In line with those studies, the reduction of the Zeta potential of APDEMS-modified solid silica nanoparticles by 2.8 mV in a 36 months stability study was reported in the NanoChOp project [34]. However, these studies suggest a relatively slow degradation, which might be overcome by immediate further modification of the amino groups (as it is done in this work) and further stabilization of the particles, as mentioned above.

## 4. Conclusions

In conclusion, uniform amino-modified porous hollow organosilica particles were synthesized successfully via the post-grafting process. The reaction parameters, i.e., pH, temperature, reaction time, and APDEMS concentration, were investigated one by one to prepare pHOP-NH_2_ with a maximum number of amino groups on the surface and a minimum degree of aggregation. The colloidal stability and surface charge for pHOP-NH_2_ samples prepared at different experimental conditions were evaluated using DLS and Zeta potential techniques. The results revealed that the concentration of amino groups on the surface could be tuned by controlling the reaction parameters. The amino surface modification of pHOPs resulted in particles whose fluorescent labelling became easily feasible. Accordingly, the fluorescent F-HOPs were prepared via covalent bonding of FITC molecules to the free amino groups on the surface of pHOP-NH_2_. The thorough characterization of F-HOPs for particle size, morphology, colloidal stability, surface charge, concentration and fluorescence properties was carried out by TEM, DLS, Zeta potential measurements, FT-IR, UV-Vis, fluorescence spectroscopies, and MRPS. The results showed that F-HOPs have a uniform spherical shape with an intact shell framework, narrow size distribution and positive surface charge at acidic pH. At the same time, the FT-IR results showed that the FITC molecules were chemically attached to the surface, which was indicated from the new bands that appeared in the FT-IR spectrum after fluorescent labelling of pHOP-NH_2_. UV-Vis and fluoresce measurements also proved the covalent surface modification: Red-shift in the adsorption spectra occurred due to the change in the molecular structure of FITC after coupling with pHOP-NH_2_. The result showed that the F-HOPs have good fluorescent properties. Thus, these particles possess physicochemical and optical properties that nominate them as suitable candidates for various flow cytometry and fluorescent endoscopy applications.

## Figures and Tables

**Table 1:** Reaction conditions for various pHOP-NH_2_ samples.

Sample	pH	Temp. (°C)	Time (min)	APDEMS (μmol)
M1	4.2	30	10	0.6
M2	7.2	30	10	0.6
M3	10.2	30	10	0.6
M4	10.2	40	10	0.6
M5	10.2	50	10	0.6
M6	10.2	60	10	0.6
M7	10.2	60	30	0.6
M8	10.2	60	60	0.6
M9	10.2	60	10	1.2
M10	10.2	60	10	1.8
M11	10.2	60	10	2.4
M12	10.2	60	10	3.0

**Table 2:** Mean diameter, PDI and Zeta potential values as obtained from DLS and Zeta potential measurements for different pHOP-NH_2_ samples.

Sample	DLS		Zeta Potential (mV)			
	Mean Dia. (nm)	PDI	pH 2.9	pH 5.1	pH 6.2	pH 8.5
pHOP	330.98	0.5	−28.1	−39.1	−42.2	−47.3
M1	386.56	0.8	−10.1	−29.8	−42.6	−47.8
M2	360.64	0.22	9.1	−13.3	−38.7	−48.9
M3	337.34	0.44	11	16.4	−43.2	−46.5
M4	325.76	0.76	13.4	11.4	−41.5	−43.3
M5	333.30	0.58	19.7	17.7	−48.6	−47.4
M6	352.90	0.33	21.2	17.7	−41.4	−44
M7	367.37	0.40	22.9	24.7	−29.1	−41.9
M8	396.38	0.12	27.4	29	−28.6	−39.8
M9	323.18	0.58	25.4	28.3	−30.4	−48.6
M10	388.25	0.06	33.3	34.7	−25.8	−47.9
M11	407.50	0.07	31	32.7	−26.5	−48.9
M12	433.73	0.15	32.3	31	−23.2	−44.2

## Data Availability

The data is available on reasonable request from the corresponding author.

## Supplementary Materials

The following supporting information can be downloaded at: https://www.mdpi.com/article/10.3390/ma15072696/s1, Figure S1: TEM image and PSD of solid SiO_2_ core particles; Figure S2: PSD and shell thickness distribution of pHOP by TEM, Figure S3: PSD and shell thickness distribution of F-HOP by TEM, Figure S4: Fluorescent emission spectra of different known FITC concentrations and calibration curve, Figure S5: SAXS curve of the F-HOP sample. Figure S6: Intensity-weighted size distribution of the F-HOP sample 3 months after preparation.
