# Supplementary material for: Amino Surface Modification and Fluorescent Labelling of Porous Hollow Organosilica Particles: Optimization and Characterization
Source: Materials (Basel). 2022 Apr 6;15(7):2696. doi: 10.3390/ma15072696 (PMC9000543; doi:10.3390/ma15072696)
Supplement: Supplementary file 1 [file materials-15-02696-s001.zip › materials-1649540-supplementary.pdf]

Supplementary Materials

Amino Surface Modification and Fluorescent Labelling of Porous Hollow Organosilica Particles: Optimization and Characterization

Mohammed A. Al-Khafaji, Anikó Gaál, Bálint Jezsó, Judith Mihály and Zoltán Varga

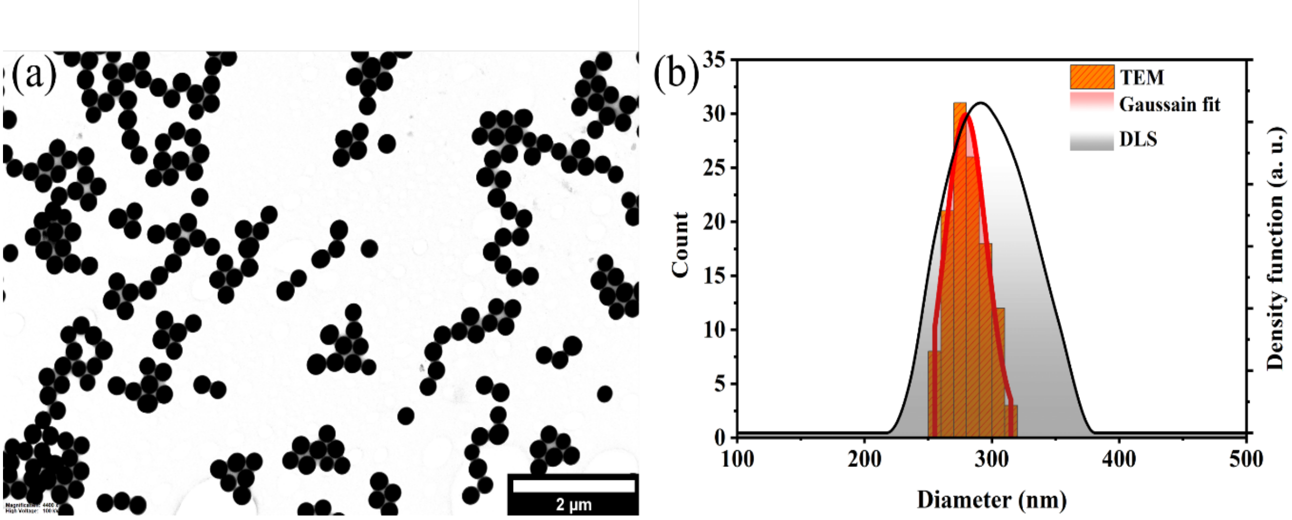

Figure S1. (**a**) TEM image and (**b**) PSD together with Gaussian fit and DLS measurements for solid SiO_2 core particles.

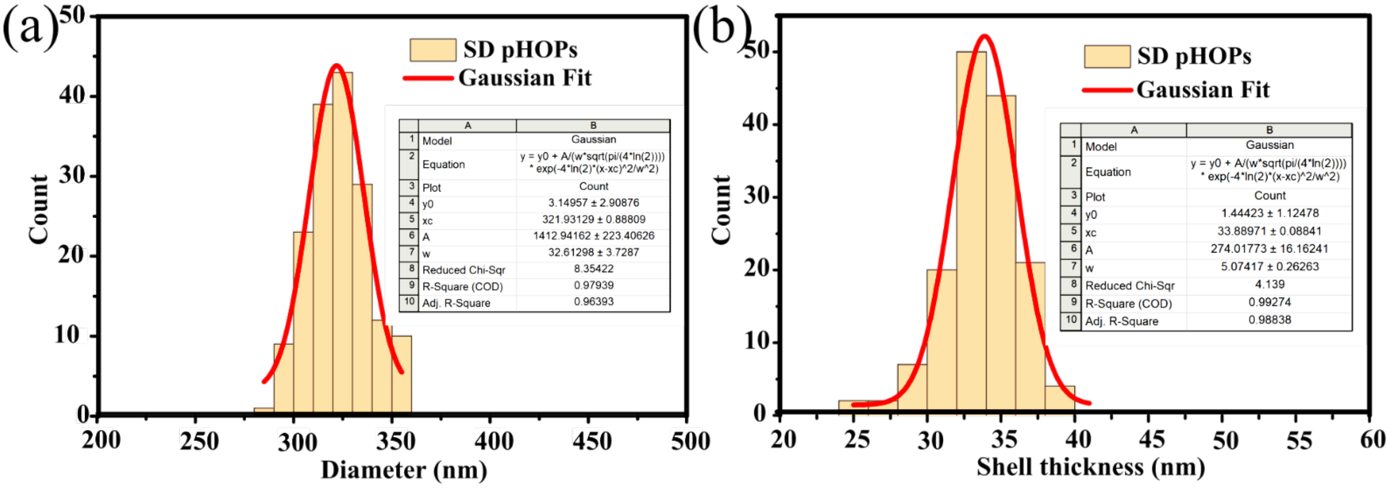

Figure S2. PSDs and Gaussian fit of porous hollow organosilica particles (**a**) and shell thickness (**b**).

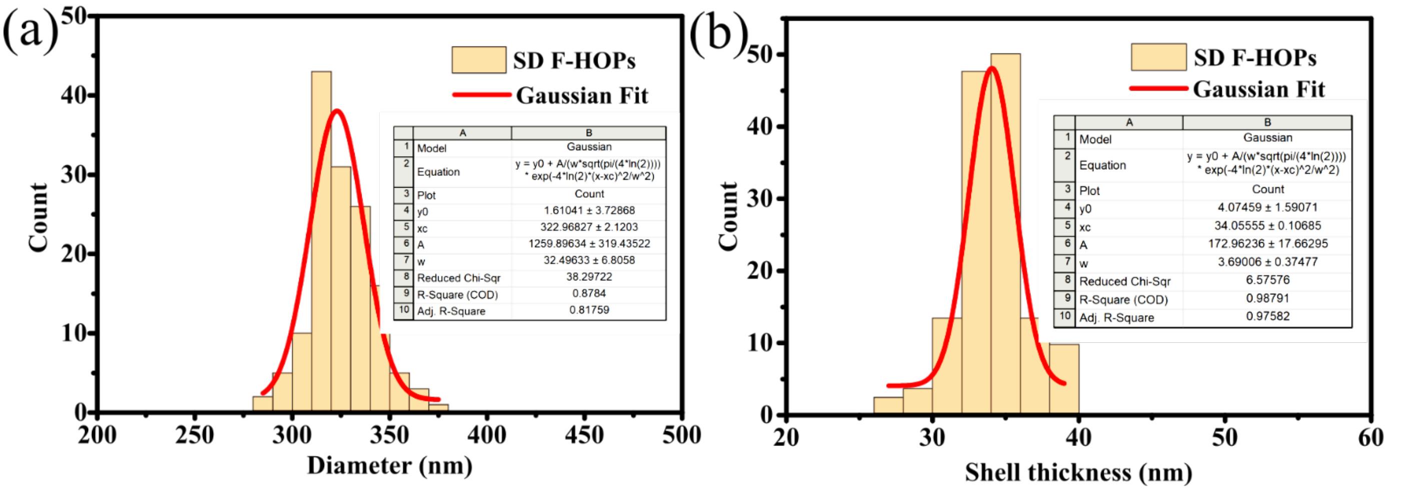

Figure S3. PSDs and Gaussian fit of florescent porous hollow organosilica particles (**a**) and shell thickness (**b**).

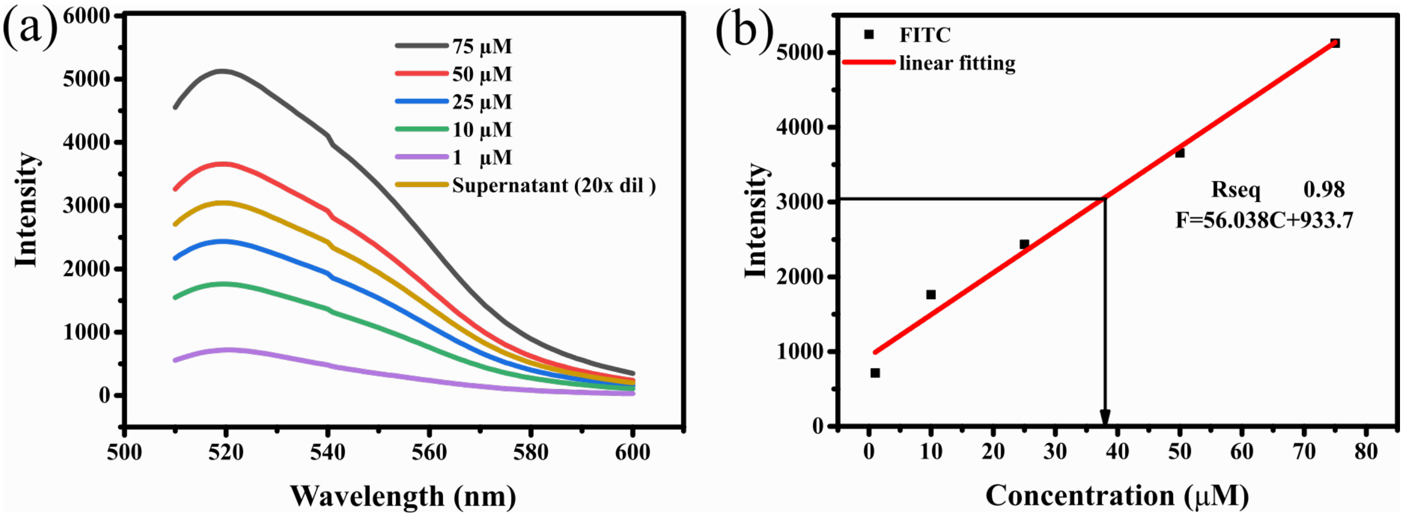

Figure S4. (**a**) Fluorescent emission spectra of different known FITC concentrations and (**b**) Calibration curve obtained by plotting the fluorescent intensities against the concentrations.

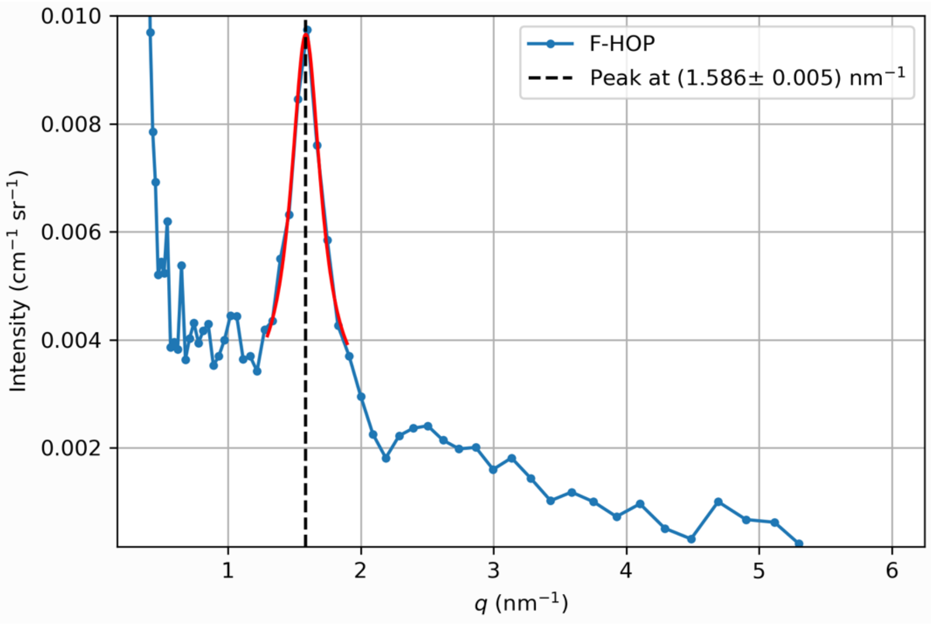

Figure S5. SAXS curve of the F-HOP sample. The characteristic peak at q = 1.586 nm^{-1} corresponds to the hexagonal order of the mesopores due to the use of CTAB as a structure-directing agent.

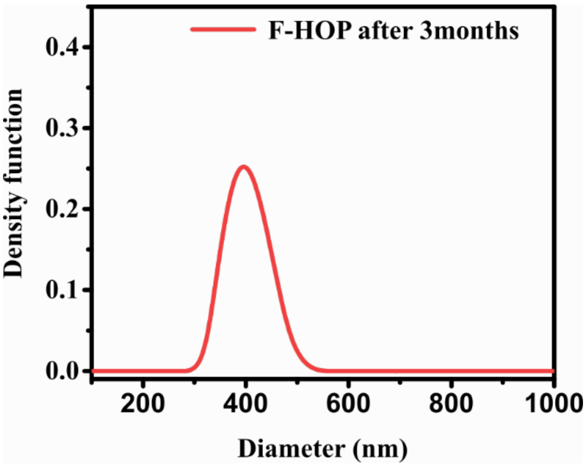

Figure S6. Intensity-weighted size distribution of the F-HOP sample 3 months after preparation measured by DLS.
